# Repair of Forearm Muscle Herniation Using Local Fascial Flap: A Case Report

**DOI:** 10.7759/cureus.4881

**Published:** 2019-06-11

**Authors:** Francisco A Schwartz-Fernandes, Alicia Lew, Melissa D Gonzalez

**Affiliations:** 1 Orthopaedics and Sports Medicine, University of South Florida Morsani College of Medicine, Tampa, USA; 2 Pediatrics, University of South Florida Morsani College of Medicine, Tampa, USA; 3 Orthopaedics, University of South Florida Morsani College of Medicine, Tampa, USA

**Keywords:** muscle herniation, fascial flap, complication cubital tunnel release, ulnar nerve transposition, muscle hernia

## Abstract

Forearm muscle herniation is a rare but known cause of symptomatic pain in the upper extremity caused by compression or strangulation of the muscle belly through a defect in the overlying fascia. Because of the rarity of this condition, optimal treatment is still widely unknown and debated. To date, there are various treatment methods published, including rest, physiotherapy, primary repair, fasciotomy, fascia lata inlay, onlay or wrap-around, mesh graft, and acellular porcine collagen matrix.

In this study, a 61-year old man underwent an ulnar nerve transposition to correct cubital tunnel syndrome, resulting in subsequent symptoms of muscle herniation on the volar aspect of the forearm. Prominent muscle herniation was visible a few weeks after the onset of symptoms and surgical correction of the fascial defect was performed using a local fascial flap. Postoperatively, the patient’s herniation symptoms resolved without signs of ulnar nerve entrapment. The rationale for this treatment option is discussed.

## Introduction

Muscle herniation is described as the bulge of a muscle through an opening or weakness of the overlying fascia that can present with focal weakness, pain, and loss of sensation. It is also believed that hypertrophied muscle tissue protruding through weak points of fascia can further exacerbate such symptoms due to the increased irritation against the fascial opening [1].

Muscle hernias are clinically uncommon conditions that can arise spontaneously from direct trauma and injury or repetitive muscle exercise. Of the cases reported, the majority occur in the lower extremity, particularly the anterior tibial compartment. Much less common and understood are upper extremity muscle herniations, which predominantly occur in the forearm flexor compartment, except one reported case of the dorsal forearm [2].

Due to the lack of published guidelines, the treatment and modes of surgical intervention are still debated and widely discussed. Multiple methods of treatment have been described, including rest, physiotherapy, direct repair, fasciotomy, fascia lata inlay, onlay or wrap-around, mesh graft, and acellular porcine collagen matrix [1, 3-6, 7-9].

Alternatively, in this report, a case successfully repaired with a local fascial flap is described.

## Case presentation

A 61-year-old Caucasian male was previously treated with an ulnar nerve transposition several years ago to treat cubital tunnel syndrome. Approximately one year later, the patient presented with symptoms of pain in the anteromedial aspect of the forearm, followed by a prominent flexor carpus ulnaris muscle herniation a few weeks later. Without any precipitating factors, such as trauma, the hernia continued to increase in size and become more uncomfortable over time. Although the patient’s cubital tunnel symptoms resolved, the herniation became troubling enough for him to seek hand surgery consultation.

On inspection, there was a clear 6 cm x 7 cm bulge through the volar aspect of the mid-forearm which became particularly evident on both active and passive flexion of the wrist with or without resistance. As a result, the decision was made to undergo surgery to repair the defect.

**Figure 1:**
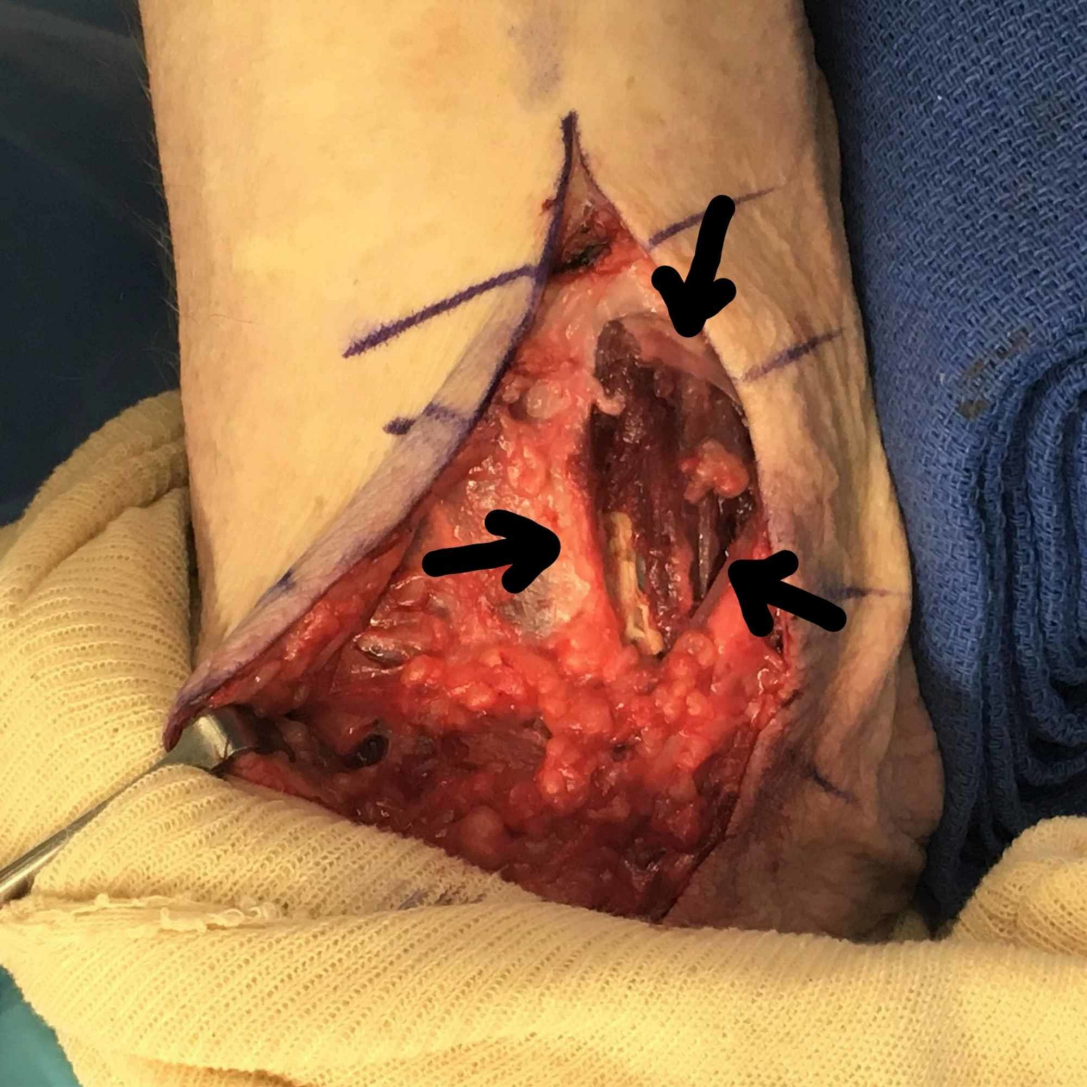
Herniation at the palmar forearm fascia on passive flexion of the left wrist Arrows indicate the herniation. The top of the picture is the distal forearm and the bottom of the picture is the proximal forearm

**Figure 2:**
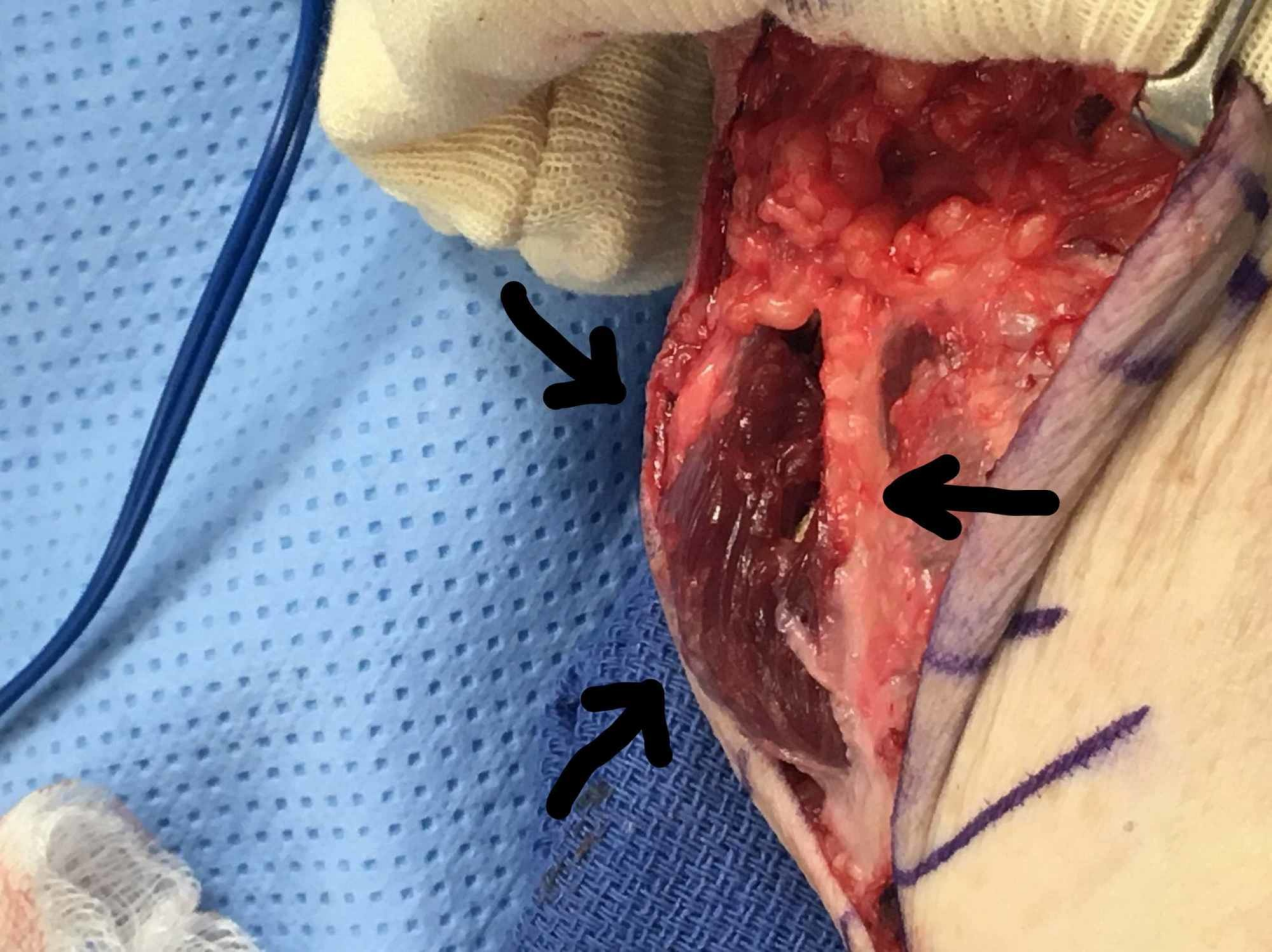
Intraoperative demonstration of a herniation at the palmar forearm fascia on passive flexion of the left wrist The top portion of the picture is the proximal forearm and the bottom portion of the picture is the distal forearm.

During surgery, a 10 cm incision was made from the medial condyle down to the end of the proximal third of the left forearm. Using blunt dissection with scissors, a 4 cm herniation was found 5 cm distal to the medial epicondyle and muscle mass of the flexor carpi ulnaris (Figures 1-2). Due to its relatively small size, a local fascial flap seemed the most appropriate course of correction.

**Figure 3:**
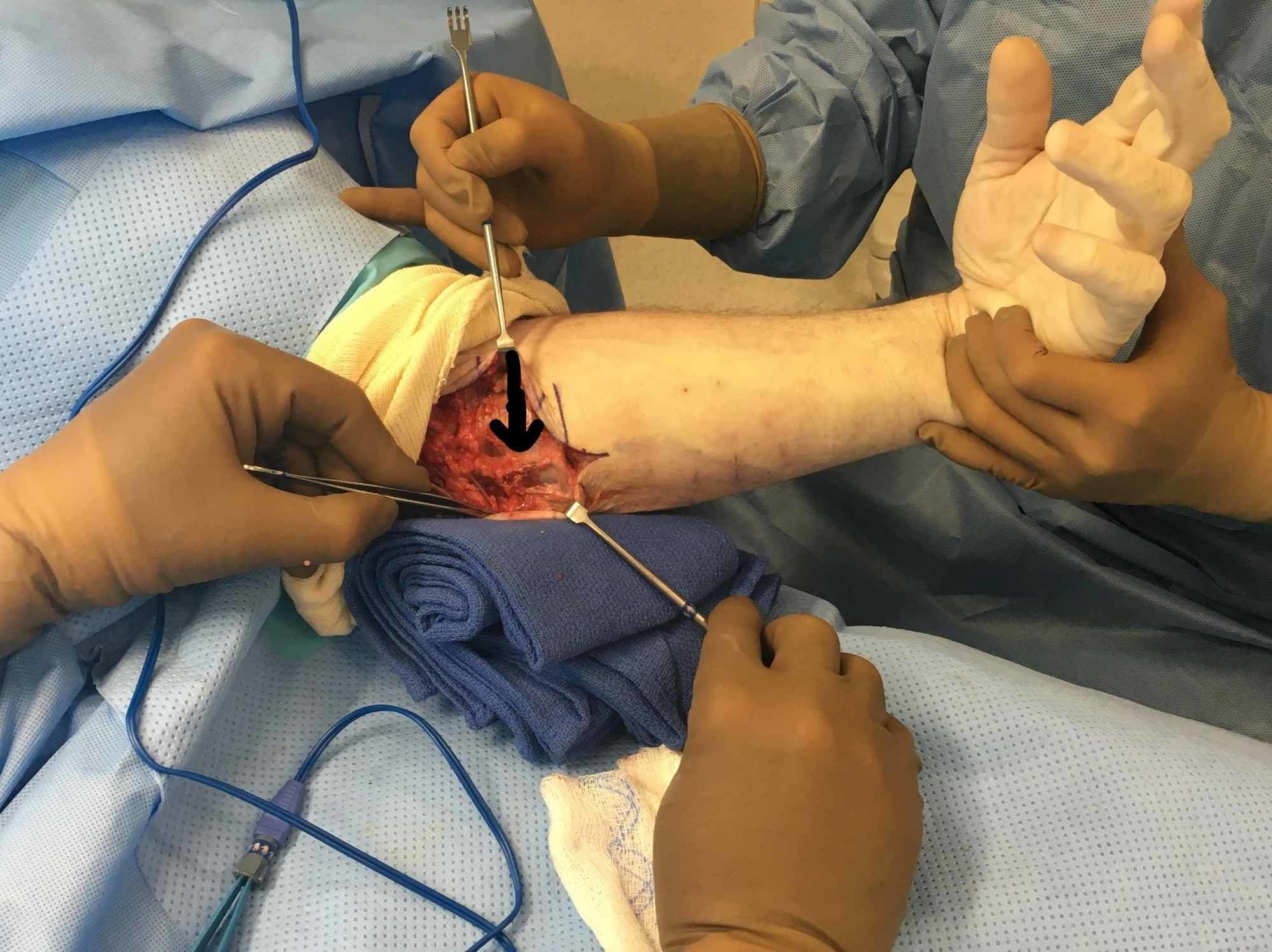
Intraoperative demonstration of the repaired hernia with the wrist in flexion Note that no more bulging is observed.

**Figure 4:**
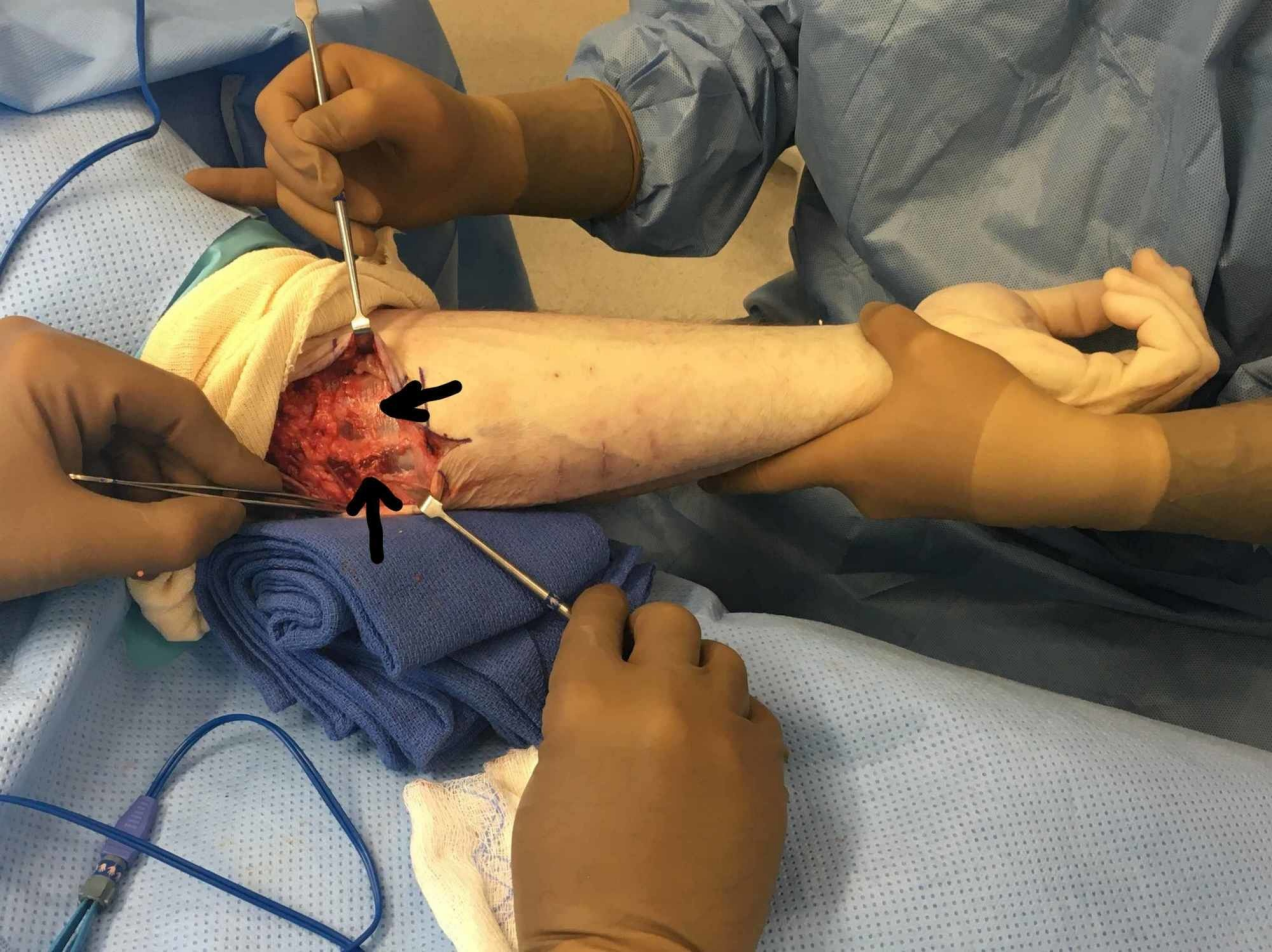
Intraoperative demonstration of the repaired hernia with the wrist in neutral position

**Figure 5:**
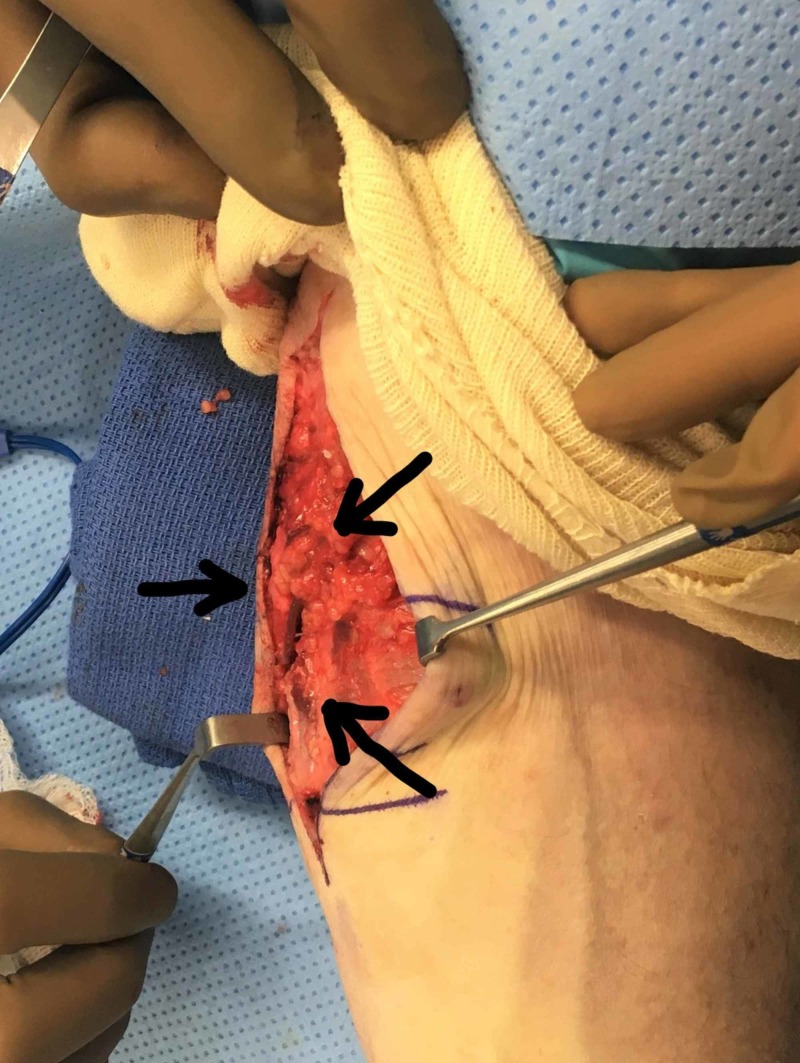
Detail of the repaired hernia with fascial flap The top portion of the picture is the proximal forearm and the bottom portion of the picture is the distal forearm.

Exploration of the flexor forearm compartment was performed to locate the ulnar nerve to prevent re-entrapment with the herniation repair. A 5 cm x 4 cm flap from the most lateral head of the flexor carpi ulnaris (FCU) was then harvested while maintaining the attachment on the lateral aspect. Next, 4-0 monofilament sutures were used to approximate the fascia from distal to proximal to completely close the defect with the fascial flap and then used to reinforce the repair. On passive flexion, the bulge was no longer visible (Figures 3-5). After the repair was completed, the patient was placed in a long arm splint with the elbow flexed for three weeks and instructed to keep the arm rested.

The patient was then seen three weeks after his surgery without any complaints or symptoms of re-herniation on active or passive flexion. The patient denied any numbness or tingling and did not have any signs of instability or tendon subluxation. His surgical site healed without any signs of infection or complications. During the last evaluation, the patient was able to flex and extend his elbow, had full pronation and supination of the forearm, and was released to medium level activity without the need of physiotherapy.

**Figure 6:**
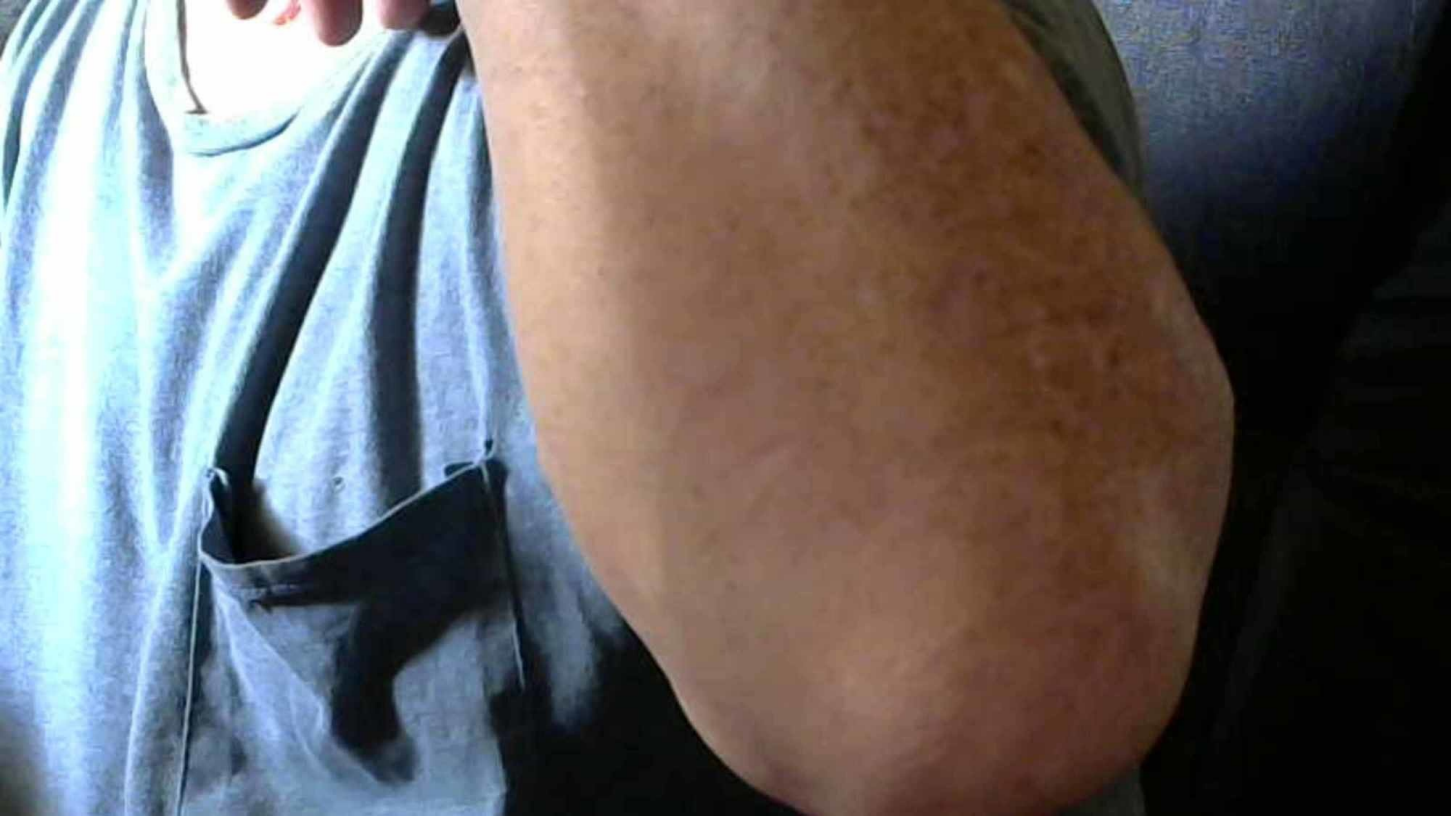
Forearm picture with the wrist in flexion and no evidence of hernia in the medial aspect of the forearm

**Figure 7:**
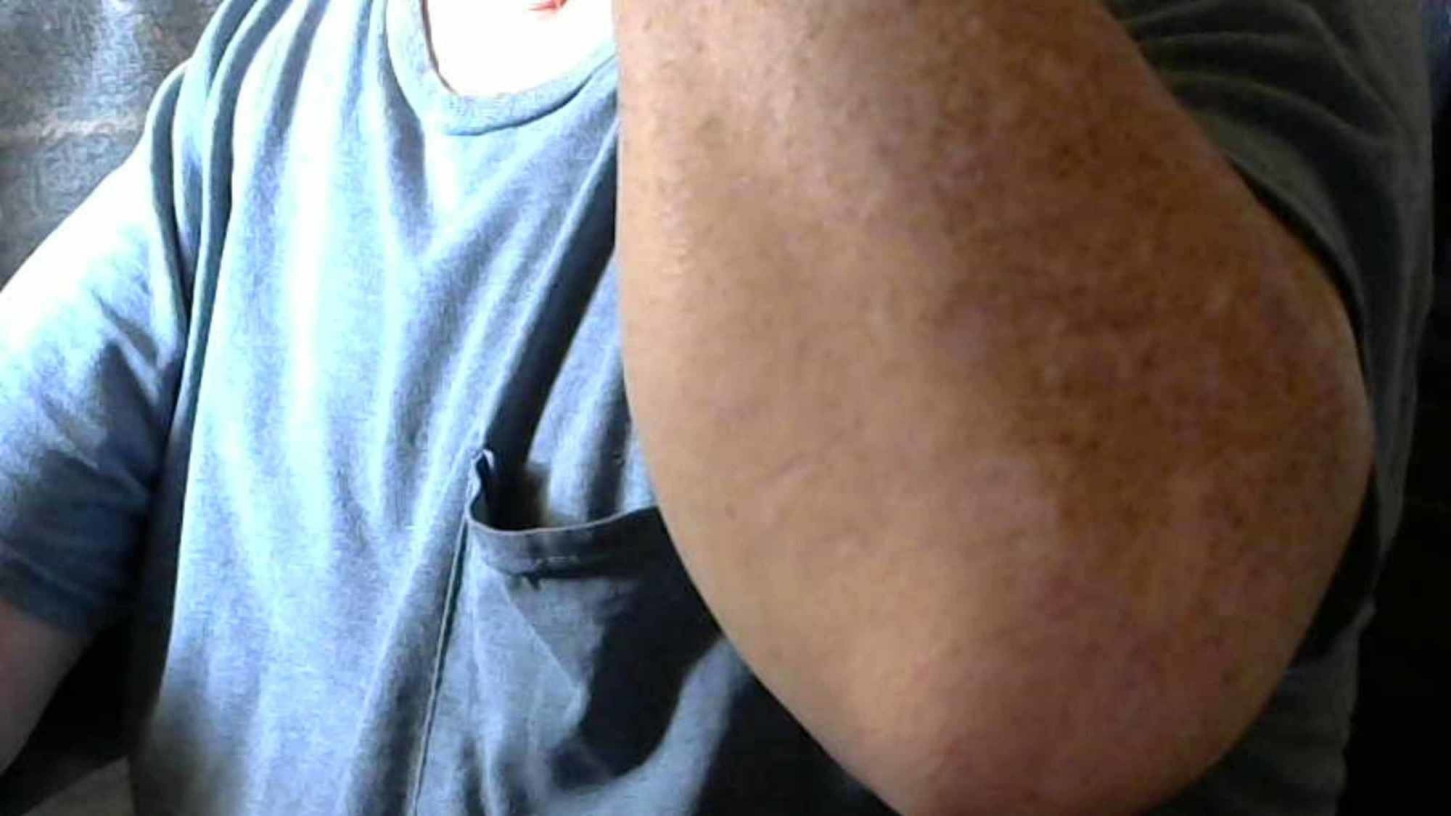
Forearm picture, wrist in extension and no evidence of hernia in the medial aspect of the left foream

At the sixth week postoperative visit, the patient returned to full activity without any neurological complaints and was happy with the aesthetic appearance of the surgical site. He was interviewed over the phone 15 months after his procedure and still did not have any complaint or recurrence of his muscular hernia. A three year follow-up confirmed that the patient was doing well. There was no recurrence of his herniation (Figures 6-7).

## Discussion

Muscle herniation is currently thought to be the result of hypertrophied muscle protruding through the weakened deep fascia, such as areas where neurovascular structures traverse across. As a result, most cases are usually due to strenuous exercise seen in athletes, military personnel, and penetrating trauma [5]. 

**Figure 8:**
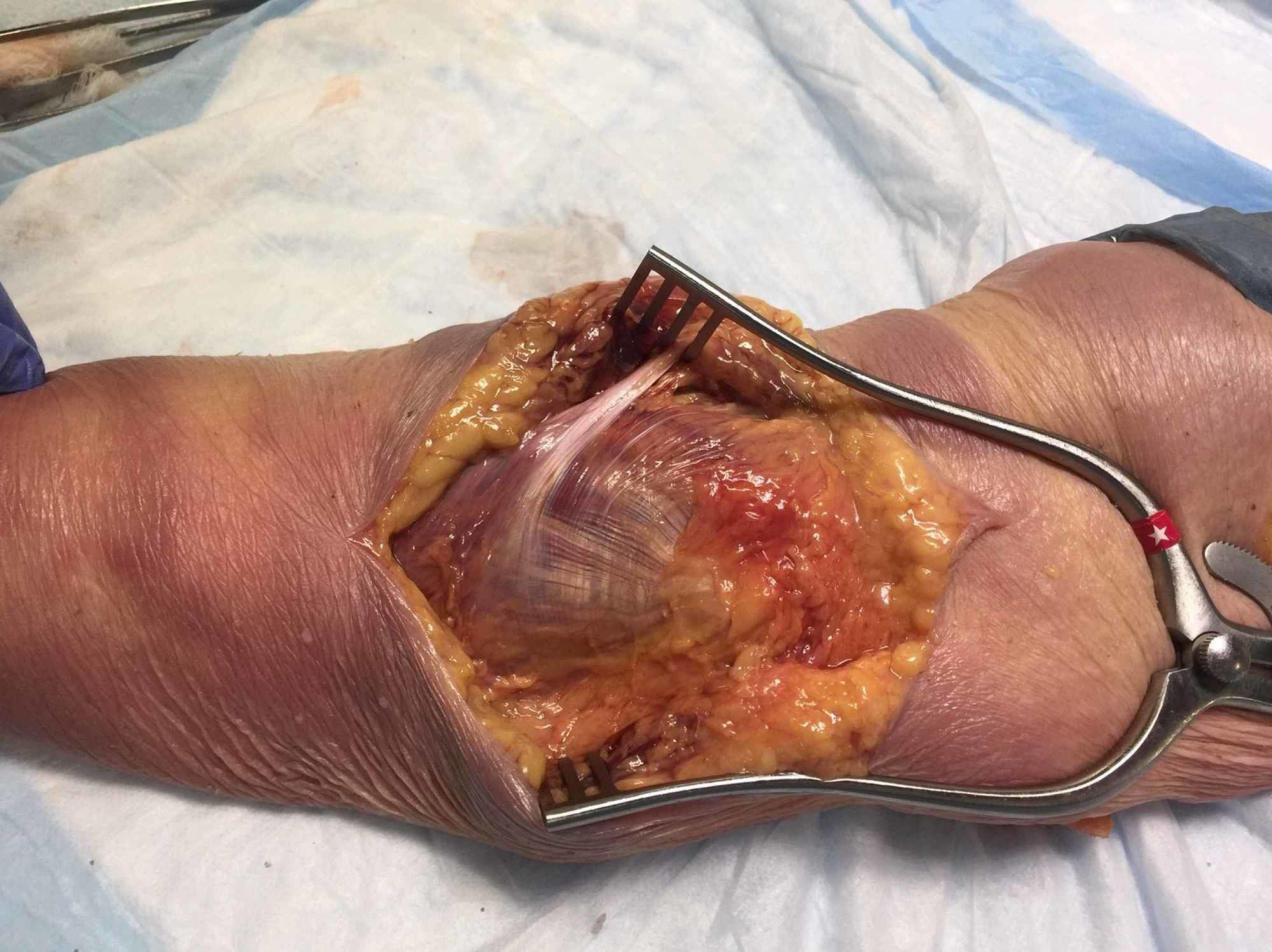
Medial aspect of the forearm fascia

**Figure 9:**
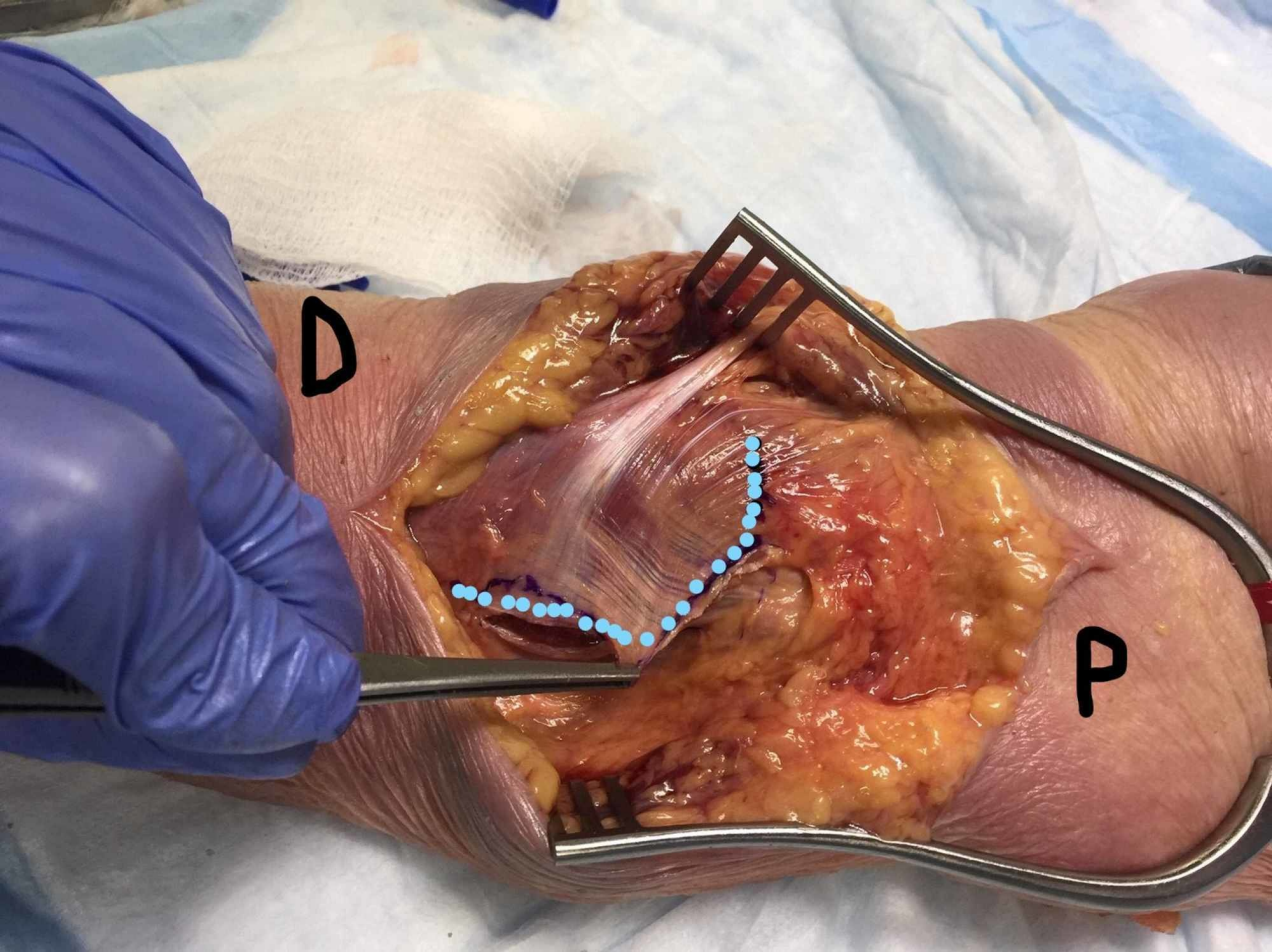
L-shaped fascial flap L-shaped fascial flap allowed the surgeon to mobilize part of the forearm fascia to cover and repair the herniation. Blue dots: site of the incision on the fascia D: distal; P: proximal

While lower extremity muscle herniations are relatively well-documented, upper extremity cases are much less reported and understood. Because of this, the optimal treatment for forearm muscle herniations is still being debated. To date, there are various treatment methods published, including rest, physiotherapy, primary repair, fascia lata inlay or wrap-around, mesh graft, and acellular porcine collagen matrix [1, 3-6, 7-10]. The goal of these treatments is to restore normal muscle fascia relationships and improve the appearance of the extremity, but complications could occur [2]. In our case, a forearm fascia flap was used, as demonstrated in the anatomical pictures below: a transverse incision across the fascia, creating an L-shaped flap, which was then mobilized medially to close the herniation (Figures 8-9).

The poorest possible complication of a muscle herniation correction is the development of compartment syndrome after direct repair of a fascial defect. This could lead to nerve damage or amputation due to loss of blood flow to the nerves and muscles [4].

Although most forearm muscle herniations reported are either due to trauma or exertional exercise, this novel case is due to surgical manipulation of the volar compartment in the transposition of the ulnar nerve. In the surgical treatment of cubital tunnel syndrome, the ulnar nerve is repositioned to prevent irritation against adjacent structures, such as the medial epicondyle and ulnar collateral ligament. However, a rare complication of this procedure, as noted in our case, was the resultant irritation of the flexor carpi ulnaris through a weakened point in the overlying fascia. 

While conservative measures, such as rest and physiotherapy, are recommended for asymptomatic herniations, surgical intervention still remains the most promising treatment for symptomatic and recurring defects [4]. 

Although Olch and Watson described six out of seven cases that were successfully treated with fasciotomy, three out of those six cases still resulted in persistent herniation or an incomplete resolution of symptoms [9]. Additionally, this procedure has been known to leave scars that are aesthetically unpleasing to patients.

Alternatively, primary repair of the fascial defect has shown some success. However, this procedure is more favorable for smaller herniations in the acute setting [6]. Additionally, this procedure performed in the lower extremity has been shown to carry a risk for anterior compartment syndrome, which should be noted when considering it for forearm herniation repair [1].

## Conclusions

In our case, we decided to choose a local fascial flap from the FCU due to its location and ease of access. This allowed for a procedure without the need for additional material or surgical sites to graft tissue for the surgical repair. The use of the patient’s tissue further avoided other risks and costs associated with synthetic mesh or autologous acellular porcine collagen matrix grafts. While using a fascia lata inlay and wrap-arounds have shown considerable success in the lower compartment, its use in the upper extremity still shows variable results. To date, our patient has successfully healed postoperatively, has returned to work, and has use of his forearm without any recurrence of symptoms or related complaints.
